# Autologous hematopoietic stem cell transplantation for multiple sclerosis: Long-term follow-up data from Norway

**DOI:** 10.1177/13524585241231665

**Published:** 2024-02-12

**Authors:** Christopher Elnan Kvistad, Anne Kristine Lehmann, Silje Agnethe Stokke Kvistad, Trygve Holmøy, Åslaug Rudjord Lorentzen, Linn Hereide Trovik, Einar Klæboe Kristoffersen, Lars Bø, Øivind Torkildsen

**Affiliations:** Department of Neurology, Haukeland University Hospital, Bergen, Norway; Haematology Section, Department of Medicine, Haukeland University Hospital, Bergen, Norway; Department of Immunology and Transfusion Medicine, Haukeland University Hospital, Bergen, Norway; Department of Neurology, Akershus University Hospital, Oslo, Norway; Institute of Clinical Medicine, University of Oslo, Oslo, Norway; Department of Neurology, Sørlandet Hospital Kristiansand, Kristiansand, Norway; Haematology Section, Department of Medicine, Haukeland University Hospital, Bergen, Norway; Department of Immunology and Transfusion Medicine, Haukeland University Hospital, Bergen, Norway; Department of Clinical Medicine, University of Bergen, Bergen, Norway; Department of Neurology, Haukeland University Hospital, Bergen, Norway; Department of Clinical Medicine, University of Bergen, Bergen, Norway; Department of Neurology, Haukeland University Hospital, Bergen, Norway; Department of Clinical Medicine, University of Bergen, Bergen, Norway

**Keywords:** Treatment response, multiple sclerosis, relapsing/remitting

## Abstract

**Background:**

Autologous hematopoietic stem cell transplantation (HSCT) is a potent treatment option for patients with aggressive relapsing-remitting multiple sclerosis (RRMS).

**Objective:**

To evaluate long-term outcomes of HSCT in MS.

**Methods:**

National retrospective single-center observational study of patients with aggressive RRMS that underwent HSCT in Norway from January 2015 to January 2018. Criteria for receiving HSCT included at least two clinical relapses the last year while on disease modifying treatment (DMT).

**Results:**

In total, 29 patients, with a mean follow-up time of 70 months (standard deviation:14.3), were evaluated. Twenty patients (69%) had sustained no evidence of disease activity (NEDA-3) status, 24 (83%) were relapse-free, 23 (79%) free of magnetic resonance imaging (MRI) activity, and 26 (90%) free of progression. Number of patients working full-time increased from 1 (3%), before HSCT, to 10 (33%) after 2 years and 15 (52%) after 5 years.

**Conclusion:**

HSCT offers long-term disease-free survival with successively increasing work participation in patients with aggressive MS resistant to DMTs.

## Introduction

Hematopoietic stem cell transplantation (HSCT) is known as a highly effective and relatively safe treatment in relapsing–remitting multiple sclerosis (RRMS) and has evolved as an attractive treatment option, especially for patients with aggressive disease. In Norway, HSCT has been reserved for patients with highly active disease despite treatment with disease-modifying therapies (DMTs). The aim of this study was to evaluate the long-term outcomes of this study population.

## Materials and methods

This was a national single-center retrospective observational study of all Norwegian RRMS patients (*n* = 30) receiving HSCT from January 2015 to January 2018. One patient (3%) did not accept study inclusion and was lost to follow-up. The study protocol, selection criteria, and the treatment protocol for the intermediate-intensity non-myeloablative regimen are described in a previous publication.^1^ Patients were followed with clinical controls at Haukeland University Hospital for approximately 3 months, at 6 months, and 1-year post-HSCT. Subsequently, the patients attended local, mostly annual neurological controls, including a 5-year follow-up control. No evidence of disease activity (NEDA-3) was defined as a composite score comprising absence of clinical relapses and sustained disability progression, in addition to no new magnetic resonance imaging (MRI) disease activity.^2^ Progression/improvement was defined as an Expanded Disability Status Scale (EDSS) score increase/decrease of 1 or more (or 0.5 if baseline EDSS was ⩾5.5).

### Statistics

Survival at different time points was estimated with Kaplan–Meier survival curves. Student’s *t* test, Mann–Whitney *U* test, and Pearson’s chi-square test were used to assess differences in clinical characteristics between patients. Statistical analyses were done with STATA version 17 (StataCorp., College Station, TX, USA).

## Results

### Patient characteristics

Mean age was 31 years, and a majority were women (76%) (Table 1). Prior to HSCT, 2 (7%) patients had used one DMT, 14 (48%) had used two, 7 (24%) had used three, and 6 (21%) had used more than three DMTs. Mean follow-up time was 70 months (standard deviation (SD): 14.3).

**Table 1.** Patient characteristics and clinical long-term outcomes.

Patient characteristic (at baseline)	
Age (years), mean (SD)	31.1 (7.3)
Gender, male:female	7:22
Age at first clinical symptom (years), mean (SD)	25.3 (6.2)
Disease duration at HSCT (years), mean (SD)	5.4 (4.1)
Total number of previous relapses, mean (SD)	5.1 (2.0)
Relapses previous year, mean (SD)	1.9 (1.0)
EDSS at baseline, mean (SD)	2.9 (1.4)
Patients with Gd-enhancing lesion on MRI previous year (%)	22/29 (76)
Numbers of previous disease-modifying therapies (%)	
1	2 (7)
2	2 (48)
3	7 (24)
>3	6 (21)
Clinical long-term outcomes	
Disease-free/NEDA-3 (%)	20 (69.0)
Relapse-free (%)	24 (82.8)
MRI event-free (%)	23 (79.3)
Progression-free (%)	26 (89.7)
EDSS stable (%)	15 (51.7)
EDSS improved (%)	11 (37.9)
Disease modifying treatments post-HSCT (%)	3 (10.3)^a^
Autoimmune diseases^b^ (%)	5 (17.2)
Symptoms of premature ovarian insufficiency^c^ (%)	13/21(61.9)
Had children (%)	5 (17.2)

SD: standard deviation; HSCT: hematopoietic stem cell treatment; EDSS: Expanded Disability Status Scale; MRI: magnetic resonance imaging; NEDA-3: no evidence of disease activity.

a One patient was treated with rituximab and two with cladribine.

b Three Graves’ disease and two hypothyreosis.

c Unknown for one patient.

### Treatment response

**Figure 1.**
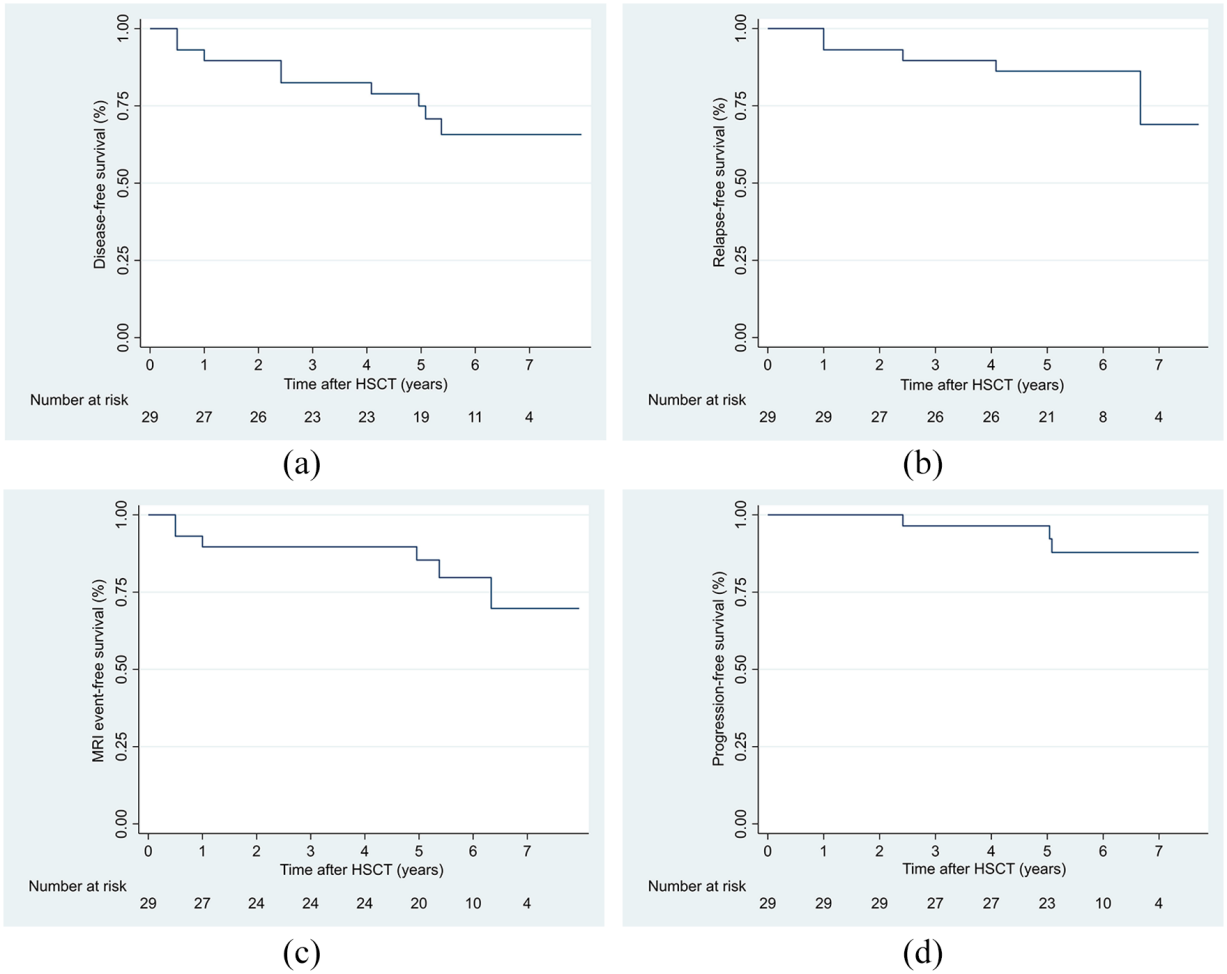
Kaplan–Meier survival curves of relapse-free survival, MRI event-free survival, progression-free survival, and disease-free survival (patient with achieved NEDA-3 status): (a) disease-free survival, (b) relapse-free survival, (c) MRI event-free survival, and (d) progression-free survival.

Overall, 20 patients (69 %) achieved NEDA-3 status (Figure 1). Patients with disease activity had significantly higher mean EDSS at baseline as compared to those without disease activity (3.7 (SD: 1.5) vs 2.4 (SD: 1.3), *p* = 0.02). Five patients (17%) had clinical relapses, six (21%) new MRI T2-lesions, and three (10%) disease progression (1.5, 2, and 3 points). A total of 11 patients (38%) had sustained EDSS improvements compared to the last scores prior to treatment, with a mean EDSS decrease of 1.5 points. The remaining 15 patients (52%) had stable EDSS. The mean EDSS difference was −0.4 points (SD: 1.2) at 5 years when compared to baseline.

### Working status

At the time of acceptance for HSCT, one patient (3%) worked full-time and five (17%) had full-time disability benefits (Table S2 and Figure S2 in the Supplemental Material). The number of patients working full-time increased to 10 (33%) at 2 years and 15 (52%) at 5 years post-HSCT. The number of patients on full-time disability benefit also increased to 10 (35%) 5 years after HSCT.

### Late adverse events and fertility

A total of five patients (17%) were diagnosed with autoimmune thyroid diseases, three with Graves’ disease, and two with hypothyroidism. Of 21 women with known fertility status, 13 (62%) experienced symptoms of persisting ovarian failure. Five patients (17%) had children after HSCT, of which two were females and three males. One of these received transplantation of ovarian tissue post-HSCT, whereas the conception occurred naturally in the other patients.

## Discussion

During a follow-up time of almost 6 years, the favorable treatment effect following HSCT was sustained. These results are consistent with those reported in other studies.^3–5^ In contrast to studies using high-dose conditioning regimens, no mortality occurred in our cohort.^6,7^ The encouraging results support the use of intermediate-intensity HSCT in RRMS and suggest that full myeloablation is not necessary for a sustained effect. A significantly higher EDSS was detected at baseline in patients not achieving NEDA-3, suggesting that HSCT is more efficient in patients with a low burden of disability, which is consistent with the results from another observational cohort study.^8^

The beneficial results were reflected in the increasingly improved working status at the end of follow-up. Patients on permanent full-time disability benefits also increased up to one-third of the population at end of follow-up, reflecting the same magnitude of patients not achieving NEDA. MS disease–associated unemployment is common, representing a substantial burden on patients as well as society.^9^ Our results are thus of interest from a socioeconomic perspective, and may be an additional argument for using HSCT in MS.

Current treatment recommendations for MS include the use of CD-20 antibodies, such as rituximab, ocrelizumab, or ofatumumab, which have demonstrated high efficacy in preventing MS relapses and new lesion formation.^10^ Our findings show that non-myeloablative HSCT represents a therapeutic alternative with similar or better response with regard to disease activity. Also, HSCT is a one-time treatment procedure, which spares the patients for long-term immunosuppression with increased risk of infections. However, HSCT has other side effects, including premature menopause and risk of thyroid disease. The positive and lasting results regarding disease activity and work participation raise the question whether more MS patients should be treated with HSCT rather than modern DMTs. Randomized clinical trials like the ongoing RAM-MS (NCT03477500), STAR-MS (NCT03477500), and BEAT-MS (NCT 04047628) will probably reveal more information, as these trials randomize RRMS patients with treatment failure to either HSCT or modern, high-intensity DMTs.

The retrospective design, lack of control group, and relatively low number of patients are limitations to this study. However, the relative scarcity of patients in this study reflects the established selection criteria for HSCT in Norway at the time of intervention.

In conclusion, our study shows that intermediate-intensity HSCT is a highly effective long-term treatment in MS, with premature post-menopause and thyroid diseases as the most frequent persisting adverse events. The initial treatment response seems to persist in the majority, offering long-term disease-free survival with successively increasing work participation for patients with aggressive MS resistant to conventional DMTs.

## Supplemental Material

sj-docx-1-msj-10.1177_13524585241231665 – Supplemental material for Autologous hematopoietic stem cell transplantation for multiple sclerosis: Long-term follow-up data from Norway Supplemental material, sj-docx-1-msj-10.1177_13524585241231665 for Autologous hematopoietic stem cell transplantation for multiple sclerosis: Long-term follow-up data from Norway by Christopher Elnan Kvistad, Anne Kristine Lehmann, Silje Agnethe Stokke Kvistad, Trygve Holmøy, Åslaug Rudjord Lorentzen, Linn Hereide Trovik, Einar Klæboe Kristoffersen, Lars Bø and Øivind Torkildsen in Multiple Sclerosis Journal

sj-docx-2-msj-10.1177_13524585241231665 – Supplemental material for Autologous hematopoietic stem cell transplantation for multiple sclerosis: Long-term follow-up data from Norway Supplemental material, sj-docx-2-msj-10.1177_13524585241231665 for Autologous hematopoietic stem cell transplantation for multiple sclerosis: Long-term follow-up data from Norway by Christopher Elnan Kvistad, Anne Kristine Lehmann, Silje Agnethe Stokke Kvistad, Trygve Holmøy, Åslaug Rudjord Lorentzen, Linn Hereide Trovik, Einar Klæboe Kristoffersen, Lars Bø and Øivind Torkildsen in Multiple Sclerosis Journal
