# Supplementary material for: Autologous hematopoietic stem cell transplantation for multiple sclerosis: Long-term follow-up data from Norway
Source: Mult Scler. 2024 Feb 12;30(6):751–4. doi: 10.1177/13524585241231665 (PMC11071593; doi:10.1177/13524585241231665)
Supplement: sj-docx-1-msj-10.1177_13524585241231665 – Supplemental material for Autologous hematopoietic stem cell transplantation for multiple sclerosis: Long-term follow-up data from Norway [file sj-docx-1-msj-10.1177_13524585241231665.docx]

**Figure 2 (supplemental). Proportion of patients with different work-status over time.**

* Includes maternity leave, students, work assessment allowance and job seekers (see table 2 supplemental)
