# Supplementary material for: Autologous hematopoietic stem cell transplantation for multiple sclerosis: Long-term follow-up data from Norway
Source: Mult Scler. 2024 Feb 12;30(6):751–4. doi: 10.1177/13524585241231665 (PMC11071593; doi:10.1177/13524585241231665)
Supplement: sj-docx-2-msj-10.1177_13524585241231665 – Supplemental material for Autologous hematopoietic stem cell transplantation for multiple sclerosis: Long-term follow-up data from Norway [file sj-docx-2-msj-10.1177_13524585241231665.docx]

**Table 2 (supplemental): Work status over time**

| Work status (%) | Baseline (n=30) | 2 years (n=30) | 5 years (n=29) |
| --- | --- | --- | --- |
|  |  |  |  |
| Full time work | 1 (3.3) | 10 (33.3) | 15 (51.7) |
| Part time work | 1 (3.3) | 0 | 4 (13.8) |
| Sick leave | 14 (46.7) | 6 (20.0) | 0 |
| Work assessment allowance | 1 (3.3) | 4 (13.3) | 0 |
| Permanent full time disability benefits | 5 (16.7) | 5 16.7) | 10 (34.5) |
| Maternety leave | 1 (3.3) | 0 | 0 |
| Student | 6 (20.0) | 4 (13.3) | 0 |
| Job seekers | 1 (3.3) | 1 (3.3) | 0 |
